# Predictive Value of Sarcopenia and Cognitive Decline in Hospitalized Older Adults

**DOI:** 10.14336/AD.2025.0418

**Published:** 2025-04-01

**Authors:** Xiaoyan Hu, Peng Sun

**Affiliations:** Center for Rehabilitation Medicine, Rehabilitation & Sports Medicine Research Institute of Zhejiang Province, Department of Rehabilitation Medicine, Zhejiang Provincial People's Hospital (Affiliated People's Hospital), Hangzhou Medical College, Hangzhou, Zhejiang, China

## To the editor

Kon-Kfir et al.'s recent study highlights a significant association between sarcopenia and cognitive decline in hospitalized older adults [1], advocating for routine assessments of sarcopenia and cognitive function upon admission. While this work addresses an important gap in acute care settings, several limitations warrant discussion to contextualize its findings and guide future research.

Unlike prior studies in community-dwelling or outpatient cohorts [2,3], this study uniquely targets hospitalized older adults—a population with higher multimorbidity and acute illness burden. This distinction is critical, as sarcopenia and cognitive impairment in hospitalized patients may reflect acute physiological stressors rather than chronic aging processes. The use of the Digit Symbol Substitution Test (DSST) diverges from traditional paper-and-pencil tools (e.g., Minimum Mental State Examination (MMSE), Montreal Cognitive Assessment (MoCA)). While innovative, this approach introduces potential biases, such as older adults' familiarity with technology, which was partially mitigated but not fully resolved by training sessions. Most longitudinal studies [4] establish temporal relationships between sarcopenia and cognitive decline. Kon-Kfir et al.'s cross-sectional design limits causal inference, a limitation absents in longitudinal cohorts.

The modest sample size (n=140) restricts power for subgroup analyses (e.g., sex-specific differences). Larger cohorts, such as those in Petermann-Rocha et al.'s meta-analysis (n≈50,000) [5], provide stronger evidence for sarcopenia's prevalence and outcomes. Excluding patients with visual impairments or terminal illnesses may underestimate the prevalence of cognitive impairment and skew results toward healthier individuals. This contrasts with studies like Hirani et al. [6], which included broader frailty criteria. While age and sex were adjusted, key confounders, such as education level, socioeconomic status, and polypharmacy, were omitted. For instance, low education correlates with both sarcopenia and cognitive decline [7], potentially inflating the observed association.

The DSST, though validated in diabetic cohorts [8], lacks evidence for hospitalized populations. Technical challenges (e.g., screen size, network issues) may compromise accuracy, unlike standardized tools like the MMSE. Conducted at one hospital, the findings may not generalize to diverse healthcare settings or ethnic groups, unlike multinational studies [9].

The study commendably calls for integrated sarcopenia and cognitive screening in hospitals. However, future work should prioritize: 1) Longitudinal Designs: To establish causality and disentangle acute vs. chronic effects. 2) Larger, Multicenter Cohorts: Enhancing generalizability and enabling stratified analyses. 3) Comprehensive Confounder Adjustment: Including education, comorbidities, and medication profiles. 4) Comparative Tool Validation: Assessing DSST against gold-standard cognitive batteries in hospitalized settings.

Addressing these gaps will strengthen the evidence base for sarcopenia and cognitive screening protocols, ultimately improving care for hospitalized older adults.
